# Incidence of Congenital Heart Disease: The 9-Year Experience of the Guangdong Registry of Congenital Heart Disease, China

**DOI:** 10.1371/journal.pone.0159257

**Published:** 2016-07-13

**Authors:** Yanji Qu, Xiaoqing Liu, Jian Zhuang, Guanchun Chen, Jinzhuang Mai, Xiaoling Guo, Yanqiu Ou, Jimei Chen, Wei Gong, Xiangmin Gao, Yong Wu, Zhiqiang Nie

**Affiliations:** 1 Department of Cardiovascular Disease Epidemiology, Guangdong Cardiovascular Institute, Guangdong Provincial Key Laboratory of South China Structural Heart Disease, Guangdong General Hospital, Guangdong Academy of Medical Sciences, Guangzhou, Guangdong, China; 2 Department of Cardiac Surgery, Guangdong Cardiovascular Institute, Guangdong Provincial Key Laboratory of South China Structural Heart Disease, Guangdong General Hospital, Guangdong Academy of Medical Sciences, Guangzhou, Guangdong, China; 3 Department of Ultrasound, Dongguan Houjie Hospital, Dongguan, Guangdong, China; 4 Department of Pediatric Cardiology, Boai Hospital of Zhongshan, Zhongshan, Guangdong, China; 5 Department of Ultrasound, Hexian Memorial Hospital, Guangzhou, Guangdong, China; University of Toronto, CANADA

## Abstract

There are 16.5 million newborns in China annually. However, the incidence of congenital heart disease (CHD) has not been evaluated. In 2004, we launched an active province-wide hospital-based CHD registry in the Guangdong Province of southern China. In this study, we examined the incidence of CHD and its subtypes from 2004 to 2012 and compared our findings to the literature. Our results indicate there is an increasing trend of CHD incidence. The increase in incidence occurred mainly for single lesion and the most common subtypes (e.g., ventricular or atrial septal defect, patent ductus arteriosus). There were no increases found for multiple lesions or more complex subtypes. The proportion of CHD cases that were detected early (e.g., 1 week) increased over time. The incidence of CHD stabilized in 2010–2012 with the average cumulative incidences of 9.7, 9.9, and 11.1 per 1,000 live births at 1 week, 1 month, and 1 year, respectively. The incidences of CHD subtypes were comparable with recent international results. The data did not support previous reports that Asian children have a higher incidence of pulmonary outflow obstructions and lower incidence of transposition of the great arteries. However, there was a lower incidence of left ventricular outflow tract obstructions observed in our series. The increase in CHD incidence observed over time was due to improved detection and diagnosis. The true incidence of CHD in China was approximately 11.1 per 1,000 live births, which is higher than previously reported.

## Introduction

China is a large developing country with a population of 1.37 billion. Additionally, China has 15% of all children worldwide. There are 16.5 million newborns in China annually [1,2]. China has made notable progress in reducing the number of child deaths as a result of its rapid economic development in recent decades. The mortality rates in neonates and children younger than 5 years old decreased by 70% (from 34.0 to 10.2 per 1,000 live births among neonates and from 64.6 to 18.5 per 1000 live births among children) from 1990–2008 [2]. The causes of death have also changed during this period. For example, the proportion of deaths from infectious causes such as pneumonia, diarrhea, and neonatal sepsis has decreased from 36% in 2000 to 22% in 2008 among children younger than 5 years old. The number of deaths from congenital abnormalities increased from 6% to 11% during the same 8-year period [2]. Congenital heart disease (CHD) has become the most common congenital abnormality and is now the leading cause of infant mortality from congenital abnormalities. The surveillance of birth defects in China showed that in 1996 CHD was the 5^th^ most common birth defect. However, CHD became the leading birth defect in 2009 [3].

Although the challenges and growing health burden of CHD are known in the medical community, the true incidence of CHD in China has not been elucidated. Most of the studies conducted after the 1980s have consistently reported the CHD incidence in China was less than 8 per 1,000 live births (Table 1) [4–15]. However, the current worldwide incidence of CHD ranges from 8 to 12 per 1,000 live births [1,16,17]. Thus, accurately identifying the incidence of CHD is important in planning the care of a potentially large number of newborns with CHD each year in China.

**Table 1:** Reported incidence of congenital heart disease (CHD) in live births, China.

Author/year	Study period	Study location	Study period after birth	Total live births	No. of CHD	Incidence per 1,000 live births
Liu [4], 1995	1987	Shanghai	<1 year	200,82	133	6.62
Li [5], 1999	1995–1997	4 cities	<42 days	47,026	130	2.76
Song [6], 2002	1996–1998	Suzhou	<42 days	12,854	67	5.21
Zhu [7], 2004	1996–2000	31 Provinces and cities	<7 days	4,437,232	3,679	0.62^a^/1.14^b^
Xia [8], 2005	1998–2003	Guangdong Province	<7 days	497,914	786	1.58
Mai [9], 2006	2000–2004	Beijing	<7 days	50,214	232	4.62
Yang [10], 2007	1992–1998	Jiaxing	<28 days	31,493	304	9.65
Yang [11], 2009	2007	Beijing	Before discharge	83,292	556	6.67
Yang [12], 2009	2007	Beijing	Before discharge	88,025	567	6.44
Chen [13], 2011	2005–2009	Beijing	<30 days	435,521	1,797	4.13
Liu [14], 2013	2007–2012	Beijing	<7 days	1,102,918	8,866	8.04
Wu [15], 2014	2008–2012	Guangdong province	<7 days	1,005,052	5,268	5.24

^a^ For 1996.

^b^ For 2000.

The literature data suggest there are geographic and ethnic differences in the birth incidence of CHD subtypes [16,17]. There are relatively more pulmonary outflow obstructions (e.g., pulmonary stenosis/pulmonary valve stenosis and tetralogy of Fallot), fewer left ventricular outflow tract obstructions (e.g., aortic stenosis/aortic valve stenosis and coarctation of aorta), and fewer transposition of the great arteries (TGA) reported in Asians compared with Western Caucasian populations [17–20]. Previous studies have suggested the variations in particular congenital heart lesions could have a biological basis and may be related to different genetic susceptibility [17,19,20].

In this study, we present the incidence of overall CHD and the major subtypes among live births from 2004 through 2012 from the Guangdong Registry of Congenital Heart Disease (GRCHD). We then compared our findings with data reported in the literature.

## Methods

### Guangdong Registry of Congenital Heart Disease (GRCHD)

GRCHD is an on-going province-wide hospital-based CHD registry in southern China. It is an active large scale surveillance system designed to study the epidemiology of CHD including stillbirths and live births throughout the region. Guangdong province is located on the southeastern coast of China. The province consists of 76,100 square kilometers and contains a permanent population of 104 million. The GRCHD was established in 2004 and had 19 surveillance sites at its inception. It gradually expanded to 39 sites in 20 cities in the province. The participating sites include primary, secondary, or tertiary-level hospitals. There are also maternal and child care centers in the local areas. Although the GRCHD sought to include hospitals/centers from all geographic areas across the entire province, only those technically qualified were enrolled. The B-mode echocardiogram was widely available after the 1980s in this region. However, neonatal and infant CHD diagnosis was established province-wide in the 1990s.

A working group was established in each registry site and consisted of personnel from various disciplines including obstetrics, pediatrics, cardiology (including cardiac surgery, pediatric cardiology, and echocardiography), radiology, and epidemiology. GRCHD formed a CHD referral network that encompasses all hospital levels to ensure priority service and reduce patient attrition and loss of follow-up. It also provides timely feedback to initiating facilities and helps improve the diagnostic skills at lower level facilities. The coordinating center for GRCHD is located in the Guangdong Cardiovascular Institute in Guangzhou, which is the capital of Guangdong province. The Institute is a major tertiary center for pediatric cardiac catheterization and surgical centers in southern China. The coordinating center oversees the registry, performs quality control, and analyzes data. The center offers annual technical and standardization training and various workshops for staff from all surveillance sites. The key echo-cardiographer from each registry site is required to undergo a 3-month in-service training and is certified by the coordinating center.

### Case ascertainment and classification

Consecutive newborns were evaluated by an obstetrician, pediatrician, or cardiologist before discharge or within 72 hours to determine whether further investigation for potential CHD was required. All neonates with cardiac murmur, cyanosis, active precordium, tachypnea (respiratory rate >60/min), diminished pulse, abnormal cardiac rhythm, feeding difficulty, or any congenital abnormality were referred for a B-mode echocardiographic examination. In addition to echocardiographic studies, some CHD patients had the diagnosis confirmed by cardiac catheterization, surgery, or autopsy. The diagnosis of every CHD case was confirmed by at least two senior cardiologists. The CHD cases were coded based on the International Classification of Diseases version 10 (ICD-10). The codes Q20-Q28 were used to classify the CHD subtypes.

This report includes data of 620,302 live births from 24 sites in 14 cities enrolled in the GRCHD (S1 Table). These sites had complete data by 2012. The neonates with a gestational age younger than 28 weeks were excluded from the study. The cases with atrial septal defects (ASD) measuring less than 5 mm, fossa ovalis, suspected patent ductus arteriosus (PDA), or patent foramen ovale were followed-up for 6 months after birth to make the final diagnosis. The cases with twins and multiple births were registered if there were more than one affected infant. All CHD cases diagnosed prior to 1 year of life were registered in the GRCHD. The total number of CHD in this report was 5,576 cases.

The CHD subtypes were classified into the following six categories: (1) left-to-right shunt (n = 6,224), (2) pulmonary outflow tract obstruction (n = 686), (3) left ventricular outflow tract obstruction (n = 177), (4) TGA (n = 266), (5) conditions with intracardiac mixing of oxygenated and deoxygenated blood (n = 261), and (6) other cardiac lesions (n = 506) (see Table 2 for the list of CHD subtypes in each category).

**Table 2:** Incidence of congenital heart disease (CHD) subtypes (per 1,000) in Guangdong Registry of Congenital Heart Disease (GRCHD) compared with European Registry of Congenital Anomalies (EUROCAT) and Hoffman’s review.

Subtypes of CHD	GRCHD 2004–2012	EUROCAT 2000–2005	Hoffman et al. 1955–2001	
	[95% CI]		Median	Upper quartile
***Left-to-right shunt***				
Ventricular septal defect	3.71 [3.56, 3.86]	3.06	2.83	4.48
Patent ductus arteriosus	3.16 [3.02, 3.30]	NA	0.57	0.78
Atrial septal defect^a^	2.89 [2.76, 3.02]	2.05	0.56	1.06
Atrioventricular septal defect	0.28 [0.23, 0.32]	0.19	0.34	0.40
***Pulmonary outflow tract obstruction***				
PS/PVS	0.69 [0.63, 0.76]	0.40	0.53	0.84
Tetralogy of Fallot	0.32 [0.27, 0.36]	0.28	0.36	0.58
Pulmonary atresia	0.09 [0.07, 0.12]	0.09	0.08	0.15
***Left ventricular outflow tract obstruction***				
Coarctation of the aorta^b^	0.13 [0.11, 0.16]	0.34	0.36	0.49
Aortic stenosis/aortic valve stenosis^b^	0.07 [0.05, 0.09]	0.14	0.26	0.39
Hypoplastic left heart syndrome^b^	0.08 [0.06, 0.10]	0.26	0.23	0.28
***Transposition of the great arteries (TGA)***				
TGA	0.43 [0.38, 0.48]	0.35	0.30	0.39
***Condition with intra cardiac mixing***				
Double outlet of right ventricle	0.28 [0.24, 0.32]	NA	0.13	0.25
TAPVC	0.10 [0.08, 0.12]	0.05	0.09	0.12
Common arterial truncus^b^	0.04 [0.03, 0.06]	0.09	0.09	0.14
***Others***				
Mitral insufficiency	0.23 [0.19, 0.27]	NA	NA	
Single ventricle	0.14 [0.11, 0.16]	0.07	0.09	0.14
Dextrocardia	0.10 [0.08, 0.13]	NA	NA	
Hypoplastic right heart syndrome	0.10 [0.07, 0.12]	0.04	0.16	0.22
Ebstein’s anomaly	0.06 [0.04, 0.08]	0.05	0.04	0.16
Persistent left superior vena cava	0.06 [0.04, 0.08]	NA	NA	
Pulmonary valve insufficiency	0.05 [0.03, 0.08]	NA	NA	
Tricuspid valve atresia^b^	0.05 [0.03, 0.06]	0.08	0.09	0.12
Mitral stenosis	0.04 [0.02, 0.05]	NA	NA	

Abbreviations: NA = Data not available; PS/PVS = pulmonary stenosis or pulmonary valve stenosis; TAPVC = total anomalous pulmonary venous connection.

^**a**^ The incidence of CHD subtype was higher in GRCHD.

^**b**^ The incidence of CHD subtype was lower in GRCHD.

### Data analysis

The CHD incidence data are presented as cases per 1,000 live births. The 95% confidential interval (CI) of the incidence was calculated assuming a Poisson distribution of CHD data. The cumulative CHD incidence was calculated at 1 week, 1 month, and 1 year after birth. The data are shown by birth year from 2004 through 2012. The time trend of cumulative 1-year incidence was displayed for single-lesion CHD versus multiple-lesion CHD. We also show the three most common CHD subtypes (ventricular septal defect (VSD), ASD, and PDA) versus the next seven common subtypes in our registry (pulmonary stenosis/pulmonary valve stenosis, TGA, tetralogy of Fallot, double outlet of right ventricle, mitral insufficiency, single ventricle, and coarctation of aorta).

The incidence of each CHD subtype in our series was compared to the data reported in a review by Hoffman et al. [16] and the report by the European Registry of Congenital Anomalies (EUROCAT) [21]. In 2002 Hoffman et al. reviewed 62 studies published after 1955. The authors estimated the median, lower, and upper quartile incidence for each CHD subtype. The authors suggested the upper quartile was the best single figure to represent the prevalence at birth of a particular CHD [16]. The upper quartile value and median were used for comparison purposes in this report. The EUROCAT working group combined data on congenital defects at birth from 43 registries in 20 European countries and reported the incidence of CHD subtypes from 2000–2005 [21].

We concluded the incidence of a specific CHD subtype in the GRCHD was greater than the value in the two major reports if the CHD incidences in EUROCAT and the upper quartile value from the review by Hoffman et al. were less than the lower limit of the 95% CI of incidence. Conversely, if the CHD incidences in EUROCAT and the median from the review by Hoffman et al. were higher than the upper limit of 95% CI in GRCHD, then we concluded that GRCHD had a lower incidence of the specific CHD subtype.

### Ethics statement

The study was conducted according to the principles expressed in the Declaration of Helsinki and was approved by the Ethics Committee of Guangdong General Hospital. All the mothers were verbally informed at admission that the hospital is obligated to report the health condition of the babies to the National Birth Defect Registry and GRCHD if the baby has a birth defect or specific cardiac defect. The clinical records were used for birth defect statistics and their personal patient information was not available to the public. There was no written informed consent obtained from the participants or kin/caregiver in the case of children. The patient information was de-identified using a composite unique ID that was created using a computerized mathematical algorithm prior to analysis. The Ethics Committee approved this consent procedure.

## Results

### Incidence of CHD and its time trends

**Fig 1:**
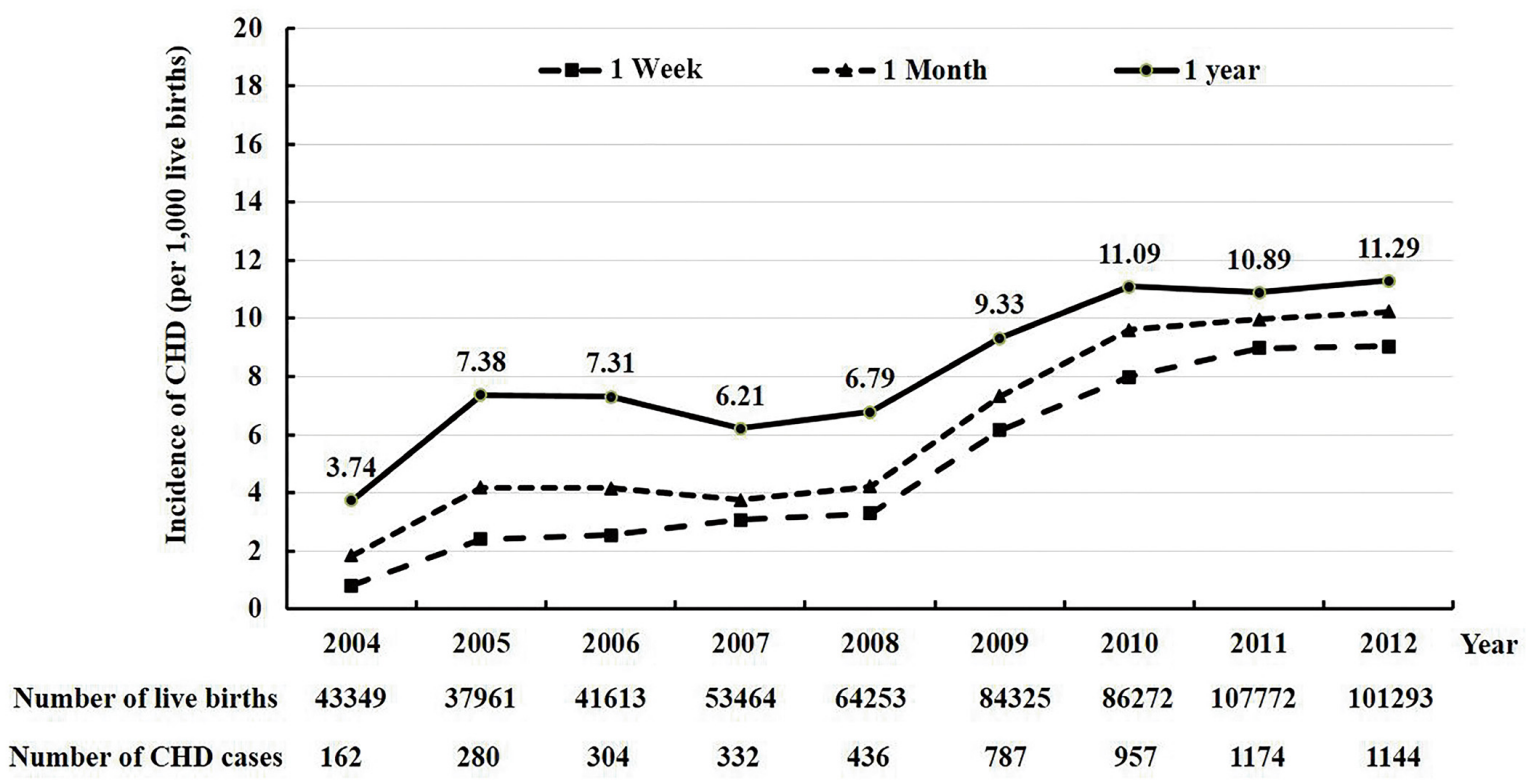
The cumulative incidence of congenital heart disease (CHD) per 1,000 live births at 1 week, 1 month, and 1 year from 2004 to 2012, Guangdong Registry of Congenital Heart Disease (GRCHD), China.

The cumulative CHD incidence data in live births at 1 week, 1 month, and 1 year after birth from 2004 to 2012 are shown in Fig 1. The three cumulative incidences increased gradually from 2004 to 2008. After 2008, the incidences increased sharply and were then stable from 2010 to 2012. During the 2004 to 2008 period, the incidence was on average 2.4, 3.6, and 6.3 per 1,000 live births at 1 week, 1 month, and 1 year, respectively. They were 8.7, 9.9, and 11.1 cases per 1,000 live births in the last 3 years from 2010 to 2012, respectively. In 2010–2012, the cumulative CHD incidence was on average 1.2 per 1,000 births higher when the observation period was extended from 1 week to 1 month and from 1 month to 1 year. Thus, an additional 15% of CHD cases were detected from 1 week after birth to 1 month of life, and an additional 28% of cases were diagnosed at 1 year of life from 1 week after birth.

**Fig 2:**
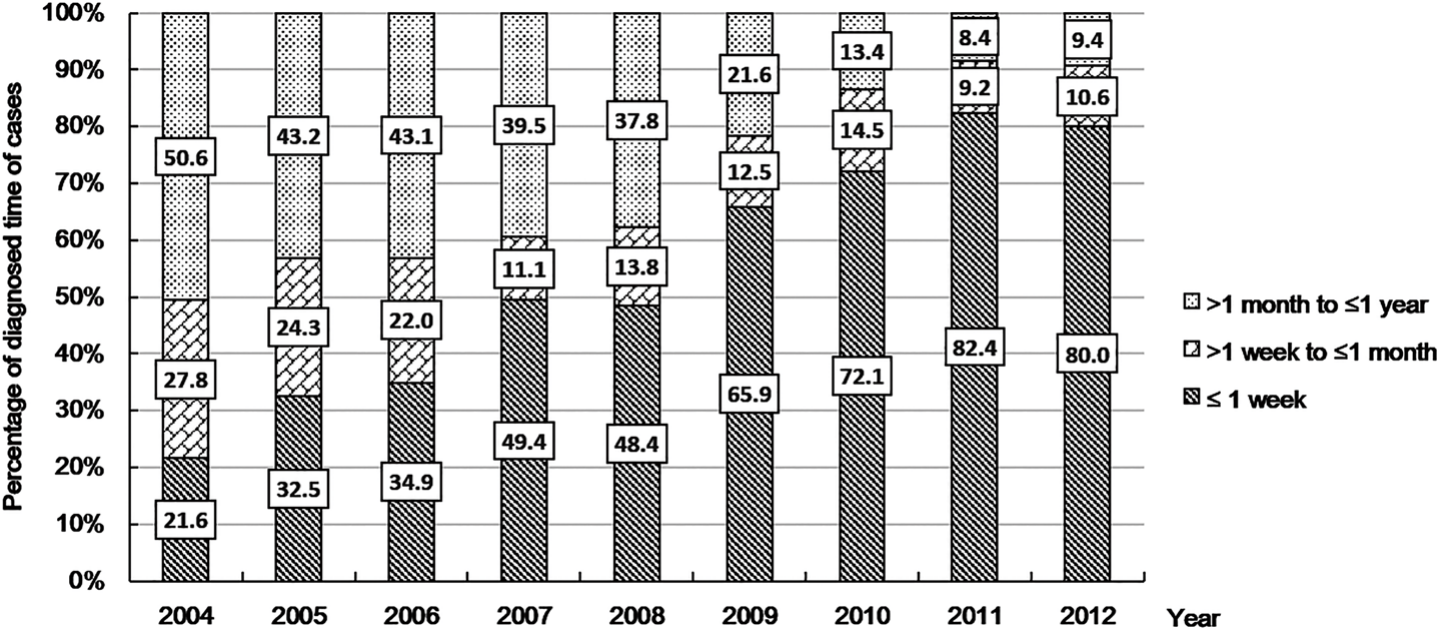
Trends of the proportion of total congenital heart disease (CHD) cases detected in 3 time periods after birth (≤1 week, >1 week to ≤1 month, >1 month to ≤1 year) from 2004 to 2012, Guangdong Registry of Congenital Heart Disease (GRCHD), China.

Fig 2 shows the trends of proportion of all CHD cases from 2004 to 2012 that were diagnosed ≤1 week, >1 week to ≤1 month, and >1 month to ≤ 1 year, respectively. There was a relatively smaller proportion of CHD cases detected early after birth (e.g., within 1 week or 1 month) during the first several years of the registry. The proportion increased consistently over time. For example, on average less than one-third of the total CHD cases were detected in less than 1 week in the period from 2004–2006. However, approximately four-fifths of the total cases were detected in less than 1 week in the period from 2010–2012.

**Fig 3:**
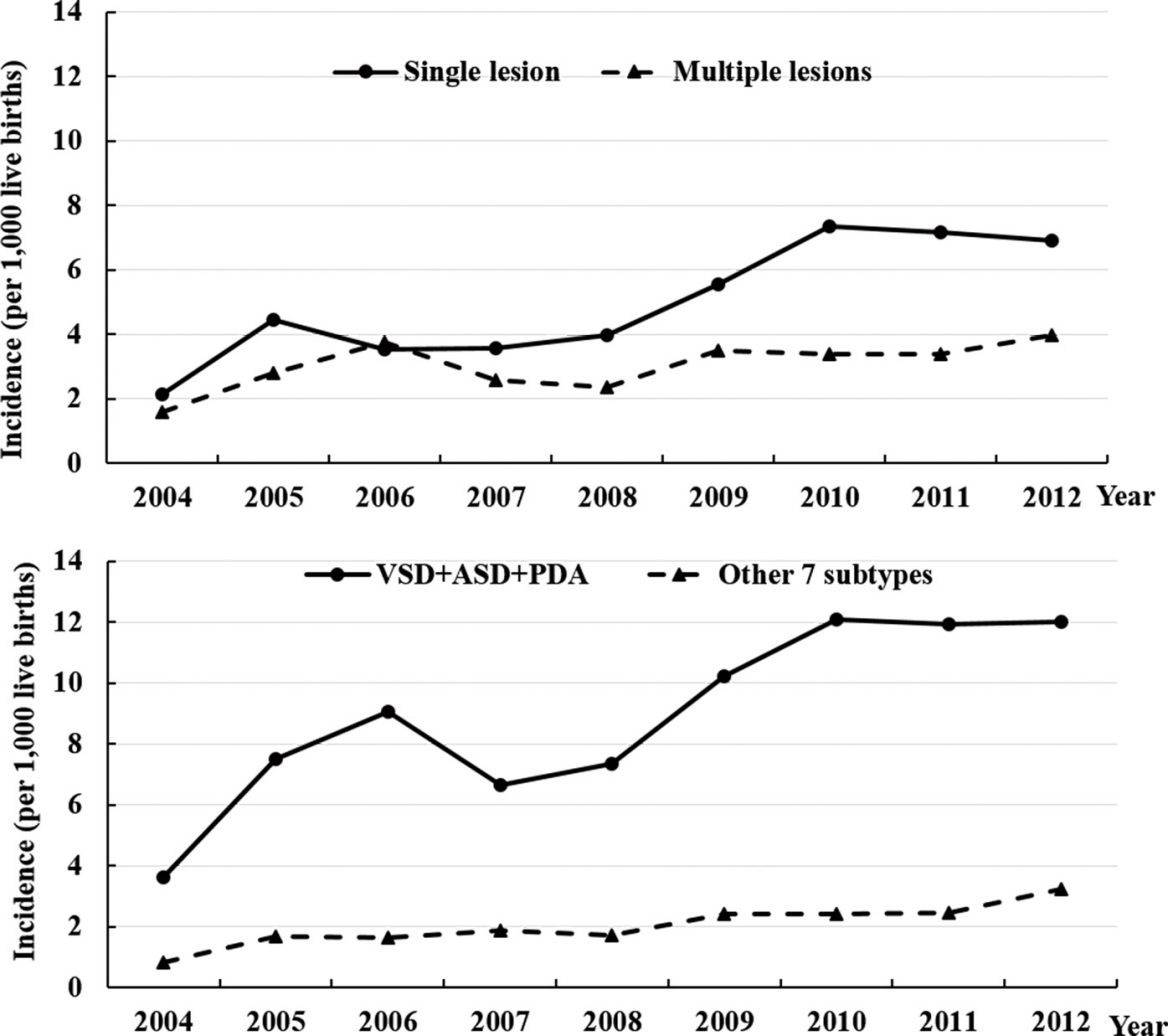
The cumulative 1-year incidence of congenital heart disease (CHD) per 1,000 live births from 2004 to 2012, Guangdong Registry of Congenital Heart Disease (GRCHD), China. Upper Panel: Single lesion vs. multiple lesions. Lower Panel: The three most common subtypes including ventricular septal defect (VSD), atrial septal defect (ASD), and patent ductus arteriosus (PDA) versus 7 other subtypes including pulmonary stenosis/pulmonary valve stenosis (PS), transposition of the great arteries (TGA), tetralogy of Fallot (TOF), double outlet of right ventricle (DORV), mitral insufficiency (MI), single ventricle (SV), and coarctation of aorta (CoA).

We examined if the increasing trend of CHD incidence was likely a “true” increase in the incidence or if this increase was associated with increased detection. We hypothesized that a “true” increase in CHD incidence was likely to demonstrate increased diagnosis of both minor defects and more complex subtypes. Fig 3 (upper panel) shows the increase in cumulative 1-year incidence of CHD occurred predominantly for single lesions and not multiple lesions. We examined the trend for the 10 most common subtypes in our registry and found two distinct groups of subtypes. There was a rapid increase in the incidence of the 3 most common subtypes and a slow increase in the incidence for the other 7 less common subtypes (lower panel of Fig 3). The first group of CHD subtypes consisted of mild cases with simple defects. The second group of subtypes contained severe cases with complex defects.

### Incidence of CHD subtypes

We compared the incidences of CHD subtypes in our registry with those reported in EUROCAT and the review by Hoffman et al. (Table 2). The most common subtype of CHD was VSD and was followed by PDA and ASD. The mean value for ASD reported by EUROCAT and the upper quartile value reported by Hoffman et al. were all less than the lower limit of 95% CI in the GRCHD. This finding indicates there is a higher incidence of ASD in the GRCHD data. The incidences of pulmonary outflow tract obstruction were not significantly different from the comparison incidences based on our predetermined criteria. However, there were lower incidences of subtypes for left ventricular outflow tract obstruction observed in the GRCHD data. The incidences of TGA, conditions with intra cardiac mixing, and various other subtypes were not significantly different from the comparison incidences with two exceptions; common arterial truncus and tricuspid valve atresia were less common in the GRCHD data. A sensitivity analysis was performed by repeating the comparisons with the data from 2010 to 2012, and the findings were similar. However, the incidence of TGA was higher in the GRCHD than in the EUROCAT and Hoffman et al.

## Discussion

The data collected during the 9 year period (2004 to 2012) of the GRCHD indicates the incidence of CHD in China may be higher than previously estimated [4–15]. The cumulative 1-week or 1-month incidence of CHD as assessed during the registry’s first several years was similar to the data reported by previous studies in China [5,7–9,13,15] and there were approximately 2–4 cases per 1,000 live births. However, the incidence then increased dramatically and stabilized in the last three years of the registry. This result reflects the “learning curve” of a large newly-established and multi-center population registry. Thus, the increase of the CHD incidence was most likely caused by improved CHD detection. Therefore, the “true” incidence of CHD may not be the average value from all years as is commonly reported in the literature [22–26] and could be more consistent with the relatively stable estimates obtained from later time points. Several registries with continuous monitoring of the same populations in Europe and the U.S. have also documented an increasing incidence of CHD over time. This increase was attributed to better diagnosis [22–24]. The improved detection and diagnostic ability in our registry were also demonstrated by the increasing proportion of all CHD cases that were diagnosed within 1 week. The increase in overall CHD incidence resulted mainly from single lesions and the most common defects. There were only small changes in the incidence of cases with multiple lesions and more complex defects. These findings are consistent with other reports [16,17,22,24–26] showing that less severe CHD that is asymptomatic has risen substantially due to improved detection. Conversely, severe CHD is often symptomatic and is easier to detect. The incidence of severe CHD has remained stable. The experience of GRCHD suggests that in a developing country the “true” CHD incidence may not be accurately determined based on data from a limited number of data points. The incidence of CHD in the latter years of our registry was approximately 11 per 1,000 live births and was comparable to the recent estimate of global incidence of CHD (8–12 per 1,000 births) by Hoffman et al. [1]. The result was higher than the estimate (9 per 1,000 births) by van der Linde et al [17].

Our study shows that longer observation periods after birth were associated with increasing case detection. The cumulative incidence of CHD was 28% higher at 1 year than at 1 week. Previous studies have reported incidences of CHD using the following detection periods: from birth hospitalization [27] to 16 years of age [26], without age limit [23], or unspecified [22,28]. Bernier et al. reviewed 35 articles and concluded that the incidence of CHD was 8 per 1,000 live births [29]. However, the diagnosis time period ranged from 1 week after birth to less than 18 years of age among cited studies. Likewise, there were large variations in the age of diagnosis among 62 studies reviewed by Hoffman et al. [16] and in the meta-analysis of 114 studies by van der Linde et al. [17]. The EUROCAT reported the average prevalence of CHD was 7.2 per 1,000 live births for 29 Europe populations in 16 European countries from 2000 to 2005 [30]. The prevalence was 6.6 per 1,000 live births for registries collecting CHD cases up to 1 month after birth. The incidence was 9.2 per 1,000 live births for registries collecting cases up to at least 1 year of life. Therefore, the impact of the observation period on the incidence estimate is substantial.

CHD is the most common birth defect and is a major cause of serious morbidity and mortality in infancy. Therefore, CHD surveillance was the focus of several population studies conducted before the 1980s [28,31,32]. CHD has been included in the surveillance of congenital abnormalities in many large multi-center registries such as the EUROCAT [21], the U.S. National Birth Defects Prevention Network [33], and the International Clearinghouse for Birth Defects Surveillance and Research [34] in the last two decades. The apparent advantage of the latter design is its ability to investigate multiple congenital defects simultaneously. However, it is also more likely to be a passive surveillance and may not focus specifically on CHD detection. The Birth Defects Monitoring Network of Guangdong Province reported the average prevalence of CHD in the first week after birth was 5.2 per 1,000 births from 2008 to 2012 [15]. The 1-week incidence of CHD year by year in the active GRCHD registry was on average 4.9 per 1,000 births higher. This comparison indicates the passive surveillance for all birth defects is likely to underestimate the true CHD incidence.

It has been suggested that compared with the reports from Western countries, children in Asian countries had a higher prevalence of pulmonary outflow obstructions and a lower prevalence of left ventricular outflow tract obstructions and TGA [17–20]. It should be noted that even in a large study there were small numbers of less common CHD subtypes, which often led to statistical uncertainty. In this report, we calculated 95% CI of the incidence for each CHD subtype in addition to the point estimate. We selected two reports with the largest numbers of CHD cases for comparisons [16, 21]. Our analyses did not show a higher incidence of pulmonary outflow tract obstruction or a lower incidence of TGA. However, there was a lower incidence of left ventricular outflow tract obstructions observed in our series. Importantly, there have been large variations in many CHD subtypes among predominantly Caucasian populations [19, 35]. For example, the prevalence of coarctation of the aorta (a subtype of left ventricular outflow tract obstruction) in 2011–2012 was as low as 1 per 10,000 birth in Odense (Demark) and 2 per 10,000 births in Paris (France). However, the incidence was as high as 6 per 10,000 in Mainz (Germany) and 11 per 10,000 in Finland [35]. Thus, it may be premature to hypothesize any genetic causes for the lower incidence of specific CHD subtypes in our population.

This study had several limitations. First, there were variations in the proficiency of CHD diagnosis among examiners. Therefore, it is possible that under-diagnosis or misclassification may have occurred. However, as a part of the quality control in GRCHD, CHD cases were referred to the coordinating center for further validations if necessary and all echocardiography reports of CHD cases were reviewed by the center. Second, we did not perform echocardiography universally on all live births and only infants with suspected CHD were examined. It is also possible that minor defects of limited importance might have been missed.

CHD is associated with a considerable disease burden for both countries and individuals. Thus, there is a need to properly identify the extent of this health problem by establishing its true incidence. The 9-year experience from the active registry GRCHD indicated that there is a learning curve for the registry. The observed increased incidence over time was likely due to improved diagnosis techniques and reporting quality for the mild CHD cases. The actual 1-year incidence of CHD was approximately 11 per 1,000 live births in Guangdong province. If the incidence was similar in other parts of China, then there would be more than 180,000 infants born with CHD each year in China, which presents a tremendous challenge for society, the health care system, and families. Although infant mortality has dramatically decreased in China in the past decades [2], there is an indication that mortality associated with CHD in infants has actually increased [36]. From 2003 through 2010, infant mortality decreased from 24 per 1,000 live births to 14 per 1,000 live births, a 42% relative reduction [37]. In contrast, infant mortality associated with CHD increased by 29% during the same time period, from 5,804 per 10 million infants to 7,471 per 10 million infants [36]. In the developed countries, a decreasing trend of infant CHD mortality have been reported consistently [38–39]. The diverse trends of overall infant mortality and infant CHD mortality in China indicates the importance of establishing good quality of CHD epidemiologic studies and surveillance systems in order to better understand the reason for this paradox. Although there is a genetic basis for CHD, the incidence of CHD can be changed. After the steady upward trend of CHD there was a decrease in prevalence of CHD in the past decade in Europe [25,40] and Canada [41]. This downward trend may be due to the intervention in maternal risk factors such as an increasing use of folic acid supplementation, better follow-up of pregnant women with chronic diseases (e.g., diabetes), and reductions in risk factors of CHD (e.g., maternal smoking). Therefore, reliable epidemiology studies of CHD in China are important. These studies will establish the true CHD burden and improve public health awareness. They also provided baseline data to evaluate the impact of interventions in reducing incidence of CHD in this large developing country that has undergone rapid demographic, economic, and environmental changes.

## Supporting Information

S1 Table Characteristics of clinical sites enrolled in the Guangdong Register of Congenital Heart Disease (GRCHD), China. (DOCX)
