# Supplementary material for: Incidence of Congenital Heart Disease: The 9-Year Experience of the Guangdong Registry of Congenital Heart Disease, China
Source: PLoS One. 2016 Jul 13;11(7):e0159257. doi: 10.1371/journal.pone.0159257 (PMC4943720; doi:10.1371/journal.pone.0159257)
Supplement: S1 Table — (DOCX) [file pone.0159257.s001.docx]

**S1 Table. Characteristics of clinical sites enrolled in the Guangdong Register of Congenital Heart Disease (GRCHD), China**

| **Regions** | **Cities** | **Population^a^** | **Number of clinical sites** | **Name of clinical sites** | **Hospital category** | **Annual births ^b^** | **Year joined GRCHD** |
| --- | --- | --- | --- | --- | --- | --- | --- |
| **Central region** | Dongguan | 8,220,237 | 1 | Dongguan Houjie Hospital | Tertiary, general | 5093 | 2004 |
|  | Foshan | 7,194,311 | 2 | Shunde Maternal and Child Care Service Centre  The 6th People’s hospital of Nanhai District, Foshan | Tertiary, specialized  Secondary, general | 7323  7080 | 2012  2011 |
|  | Guangzhou (urban) | 12,700,800 | 2 | Guangdong General Hospital  The Second Affiliated Hospital of Sun Yat-sen University | Tertiary, general  Tertiary, general | 2448  1286 | 2007  2004 |
|  | Gungzhou (suburban) |  | 4 | Panyu Maternal and Child Care Service Centre  Panyu General Hospital  Conghua Central Hospital  Huadu Maternal and Child Care Service Centre | Secondary, specialized  Tertiary, general  Secondary, general  Secondary, specialized | 4900  3742  2869  4369 | 2004  2004  2004  2004 |
| **South region** | Jiangmen | 4,448,871 | 1 | Jiangmen Maternal and Child Care Service Center | Secondary, specialized | 3811 | 2004 |
|  | Shenzhen | 10,357,938 | 2 | Baoan Maternal and Child Care Service Centre  Shenzhen Maternal and Child Care Service Centre | Secondary, specialized  Tertiary, specialized | 8349  16239 | 2007  2012 |
|  | Zhongshan | 3,120,884 | 1 | Boai Hospital of Zhongshan | Tertiary, specialized | 5549 | 2004 |
|  | Zhuhai | 1,560,229 | 1 | Zhuhai Maternal and Child Care Service Center | Tertiary, specialized | 4922 | 2008 |
| **North region** | Qingyuan | 3,698,394 | 1 | Yingde Maternal and Child Care Service Center | Primary, specialized | 1665 | 2004 |
|  | Shaoguan | 2,826,612 | 1 | Shaoguan Maternal and Child Care Service Center | Secondary, specialized | 1223 | 2004 |
| **West region** | Maoming | 5,817,753 | 1 | Huazhou General Hospital | Secondary, general | 3827 | 2004 |
|  | Yangjiang | 2,421,812 | 2 | Yangchun General Hospital  Yangchun Maternal and Child Care Service Center | Secondary, general  Secondary, specialized | 1719  2373 | 2004  2004 |
|  | Zhanjiang | 6,993,304 | 2 | Zhanjiang Maternal and Child Care Service Center  Lianjiang Maternal and Child Care Service Center | Secondary, specialized  Primary, specialized | 3504  4424 | 2009  2004 |
|  | Zhaoqing | 3,918,085 | 2 | Zhaoqing General Hospital  Zhaoqing Maternal and Child Care Service Centre | Tertiary, general  Secondary, specialized | 1963  1724 | 2010  2004 |
| **East region** | Shanwei | 2,935,717 | 1 | Haifeng Pengpai Memorial Hospital | Secondary, general | 2199 | 2011 |
| **Total** | 14 cities | 76,214,947 | 24 | Central 9, South 5, North 2, West 7, East 1. | Tertiary 9, Secondary 13, Primary 2. | 102,601 |  |

^a^ From the 6^th^ national population census in 2010-2011.

^b^ Average of the reported years.
